# Quantification of Epstein-Barr virus DNA load, interleukin-6, interleukin-10, transforming growth factor-β1 and stem cell factor in plasma of patients with nasopharyngeal carcinoma

**DOI:** 10.1186/1471-2407-6-227

**Published:** 2006-09-24

**Authors:** Eng-lai Tan, G Selvaratnam, R Kananathan, Choon-kook Sam

**Affiliations:** 1 School of Pharmacy and Health Sciences, International Medical University, Bukit Jalil, 57000 Kuala Lumpur, Malaysia; 2 NCI Cancer Hospital, Jalan BBN 2/1, 71800 Nilai, Negeri Sembilan, Malaysia; 3 NPC Laboratory, Institute of Postgraduate Studies, University of Malaya, 50603 Kuala Lumpur, Malaysia

## Abstract

**Background:**

Nasopharyngeal carcinoma (NPC) is a common epithelial neoplasm among the Chinese populations in Southern China and South East Asia. Epstein-Barr virus (EBV) is known to be an important etiologic agent of NPC and the viral gene products are frequently detected in NPC tissues along with elevated antibody titres to the viral proteins (VCA and EA) in a majority of patients. Elevated plasma EBV DNA load is regarded as an important marker for the presence of the disease and for the monitoring of disease progression. However, other serum/plasma parameters such as the levels of certain interleukins and growth factors have also been implicated in NPC. The objectives of the present study are, 1) to investigate the correlations between plasma EBV DNA load and the levels of interleukin (IL)-6, IL-10, TGF-β1 and SCF (steel factor) and 2) to relate these parameters to the stages of NPC and the effect of treatment.

**Methods:**

A total of 78 untreated NPC patients were enrolled in this study. Of these, 51 were followed-up after treatment. The remaining patients had irregular or were lost to follow-up. Plasma EBV DNA was quantified using real-time quantitative PCR. The levels of plasma interleukins and growth factors were quantified using ELISA.

**Results:**

A significant decrease in EBV DNA load was detected in plasma of untreated NPC patients (1669 ± 637 copies/mL; n = 51) following treatment (57 ± 37 copies/mL, p < 0.05); n = 51). Plasma EBV DNA load was shown to be a good prognosticator for disease progression and clinical outcome in five of the follow-up patients. A significant difference in IL-6 levels was noted between the untreated patients (164 ± 37 pg/mL; n = 51) and following treatment (58 ± 16 pg/mL, p < 0.05; n = 51). Positive correlations between EBV DNA load and IL-10 (*r*(49) = 0.535, p < 0.01), between IL6 and IL-10 (*r*(49) = 0.474, p < 0.01) and between TGF and SCF (*r*(49) = 0.464, p < 0.01) were observed in patients following treatment. None of the parameters tested including IgA-VCA were associated with tumour stages.

**Conclusion:**

We conclude that among the parameters investigated, EBV DNA load and IL-6 levels were promising markers for the presence of NPC and for the assessment of treatment outcome.

## Background

Nasopharyngeal carcinoma (NPC) is rarely reported in the West but occurs in high frequencies in Southern China, Hong Kong, Taiwan, Singapore and Malaysia [1,2]. In Malaysia for the year 2003, NPC was the second major cause of cancer mortality among males and ranked twelfth among females. The frequencies NPC in males and females were, respectively, 8.8% and 2.5% of the total cases of cancers reported. NPC is an epithelial neoplasm arising from the fossa of Rosenmuller situated at the post-nasal space [3]. NPC is classified into three histological types with the WHO Type II being more prevalent in Asia and is strongly associated with Epstein-Barr virus (EBV) [4]. EBV has been implicated as an important aetiological agent for NPC with most patients demonstrating elevated levels of IgA to the viral capsid antigen (VCA) and early antigens (EA). A serum profile that shows elevated antibody titres to these viral proteins usually presents a significant positive diagnosis for the presence of malignancy [5]. Cell-free EBV DNA has been shown to be a promising prognosis marker especially for the monitoring of NPC. Using real-time quantitative PCR, plasma EBV DNA was found to be significantly elevated in up to 96% of NPC patients compared to only 7% in the controls [6]. There have been scanty but emerging reports on the importance of immuno-modulatory cytokines and growth factors in NPC. Most of these investigations attempted to relate the expression of cytokines and their respective receptors to the outcome and prognostication of NPC [7-9]. The knowledge on the expression and functions of certain cytokines in relation to NPC might be able to explain the lack of an effective tumour-specific immune response despite the induction of EBV-specific humoral and cytotoxic response in NPC patients. Some of the cytokines and growth factors that are known to be important in NPC include interleukin (IL)-6, IL-8, IL-10 and transforming growth factor, beta-1 (TGF-β1). It would be interesting to reconcile the presence of plasma EBV DNA to the levels of these cytokines and growth factors and to determine if there are any associations between these parameters and the status of disease.

This present study investigated the concentrations of five plasma factors – EBV DNA, IL-6, IL-10, TGF-β1 and SCF (steel factor) – in NPC patients before and following treatment. The data obtained indicated a discrepancy in the levels of these factors in NPC patients before and after treatment and in relation to healthy individuals.

## Methods

### Biological and clinical samples

The Namalwa cell line was cultured in RPMI-1640 supplemented with 10% (v/v) FCS and 300 mg/L L-glutamine. The genome of each cell contains two integrated copies of the EBV episomal DNA. Total DNA extracted from the Namalwa cells was used as quantitative standards in the real-time PCR assay. A conversion factor of two EBV copies per 6.6 pg of genomic DNA was used.

Peripheral blood was collected in a tube containing EDTA to facilitate the subsequent separation of plasma. Blood samples were collected from 78 untreated, pathologically confirmed NPC patients when they first presented at the NCI Cancer Hospital from January 2003 to January 2005 after informed consent was obtained. Tumours were staged according to the AJCC/UICC (1997) classification system. Samples were also collected from 51 of the 78 patients following treatments consisting of radio- and chemotherapy. As controls, 39 blood samples from age and sex-matched healthy individuals were obtained from the University of Malaya Medical Centre (UMMC) organ matching laboratory. Plasma isolation was performed for each sample immediately upon receipt in the laboratory, aliquoted and stored at -20°C until use. Plasma samples of patients and controls were titrated for the IgA anti-EBV VCA antibody using indirect immunofluorescence (IFA). This study was approved by scientific and ethical committees of NCI Cancer Hospital and UMMC respectively.

### DNA extraction

DNA was extracted from plasma samples and the Namalwa cells using the QIAamp^® ^DNA minikit (Qiagen, USA) following the manufacturer's instructions. A 200 μL aliquot of plasma from each sample or suspension containing 1.0 × 10^7 ^Namalwa cells was used for DNA extraction. From this point onwards, all samples were processed according to the manufacturer's instructions. The extracted DNA was quantified and checked for purity using a spectrophotometer (Shimadzu, Japan).

### Real-time quantitative PCR

Quantification of EBV DNA load in plasma was performed using the iCycler iQ™ Real-time PCR system (Bio-Rad, USA). The quality of purified DNA from plasma samples was validated by conventional PCR amplification of the humanβ-globin gene using gene-specific primers (βF: 5'-AGGAGTGGTGGCTCATGTCT-3' andβR: 5'-CTCAAGGGATCCTCCCATTT-3'). Primers flanking the *Bam*H1W region (EBV coordinate: 14649–14724) of the EBV genome and TaqMan^® ^probe (Applied Biosystems, USA) directed within this flanked region (EBV coordinate: 14672–14698) were reported by Lo and co-workers [6] and were custom-made (Applied Biosystems, USA). An aliquot of five μL of purified DNA isolated from the plasma was used for amplification in a total reaction volume of 50 μL, which contained the following components; 300 nM of each primer, 25 nM of TaqMan^® ^probe and TaqMan^® ^PCR Reagents consisting of 4 mM MgCl_2_, 200 μM of each dATP, dCTP and dGTP; 400 μM of dUTP; 0.5 U AmpErase uracil N-glycosylase (UNG) and 1.25 U AmpliTaq Gold (Applied Biosystems, USA). Amplification reaction for each sample and standard was performed in duplicates. The standard curve correlating the viral DNA copy to threshold cycle was constructed by amplifying five μL aliquots of serially diluted DNA isolated from Namalwa cells that contained 45, 450, 45000, 100000 and 450000 EBV DNA copies per mL. To assess the inter-assay reproducibility of our RTQ-PCR assay, the Namalwa DNA standards in five logs of concentration as mentioned were amplified in triplicates on four separate days.

Amplification cycling was initiated by incubation at 50°C for 2 minutes for UNG activation and initial denaturation at 95°C for 8 minutes, followed by 40 cycles of 95°C for 30 seconds and 56°C for one minute. A standard curve was only accepted if the correlation coefficient is 0.996 or higher and its slope ranged between -3.74 to -3.32, which correlated to amplification efficiency of between 85 to 100% respectively. The fluorescence detection threshold value was set at 10× the mean standard deviation of fluorescence in all reactions. EBV DNA load, expressed as viral copy number per mL of plasma, was determined using the equation below.

### EBV DNA copy/mL = Q (VE/VA) *1/VP

Q: DNA copy determined from standard curve; VE: Volume of DNA eluent (50 μL); VA: Volume of DNA template amplified (five μL); VP: Volume of plasma used for DNA extraction (200 μL).

### Indirect Immunofluorescence Assay (IFA)

The expression of VCA was induced in P3HR-1 cells using 20 ng/ml of 12-*O*-tetradecanoyl phorbol 13-acetate (TPA, Sigma) and 3 mM sodium butyrate (NBA, Merck). The induced P3HR-1 cells were fixed on Teflon-coated multi-wells slides in cold acetone and dried. The fixed cells in each well was incubated with a serially diluted serum sample and later, challenged with fluorescein-conjugated goat anti-human IgA antibody that allowed indirect detection of VCA-specific antibody using a UV-microscope (200× magnification). A sample was considered IgA-VCA positive when more than 10% of the induced P3HR-1 cells showed specific fluorescence. Non-specific fluorescence staining was cross-checked with the EBV-negative BJAB cells. Negative and positive sera with respect to VCA were included in every assay.

### Enzyme-linked immunosorbent assay (ELISA)

The concentrations of IL-6, IL-10, activated TGF-β1 and SCF in the plasma were determined using the BD OptEIA ELISA Set (BD Biosciences, USA) for each individual cytokine and growth factor following the manufacturer's protocol. Briefly, a 96-well flat bottom Nunc Maxisorp™ (Nalge Nunc Intl., USA) was coated with capture antibody specific to the cytokine or growth factor of interest. The plate was washed and blocked before 100 μL of plasma and serially diluted specific standards were added to the respective wells. Following a series of stringent washing, the captured cytokine or growth-factor was detected using the specific HRP-conjugated detection antibody. The substrate reagent was added into each well and following colour development, the plate was read at 630 nm wavelength using an ELISA plate reader.

### Statistical analyses

All statistical analyses were preformed using the program, SPSS (Statistical Program for Social Sciences; SPSS Inc, USA). The means of different plasma cytokine/growth factor levels and EBV DNA load in untreated and treated NPC patients and healthy control was compared using One-way ANOVA. Bonferroni and Dunnett C methods were employed as the post-hoc tests when the means between the groups of interest were found to be significant by the ANOVA *F *test. Paired-sample *t *test was used to compare the means of cytokine levels and EBV load in NPC patients before and after treatment. Pearson correlations were used to determine the relationship between EBV DNA load and different cytokine/growth factor levels. Bivariate linear regression was employed in assessing the associations between plasma EBV load and cytokine levels (as the dependent variables), and tumour stage and IgA-VCA titres (as the independent variables). ANOVA *F *and *t *tests were used to determine the significance of the associations between the independent and dependent variables.

## Results

### Tumour stages and EBV serology

Tumours were staged according to the AJCC/UICC (1997) classification system. The distribution of the 78 untreated NPC patients according to tumour stages was as follows: five stage I, two stage IIA, 17 stage IIB, 14 stage III, one stage IIIB, 19 stage IVA, 19 stage IVB and one stage IVC. Due to significant disparity in the number of patients in different tumour subgroups, patients belonging to subgroups of a particular stage were combined forming only four major groups consisting of stages I, II, III and IV. All patients were seropositive for EBV with IgA-VCA titres ranging from 5 to 80 (median titre = 20), whereas all the 39 controls had IgA-VCA titres < 5.

### Plasma EBV load, tumour stages and IgA-VCA titres

**Figure 1:**
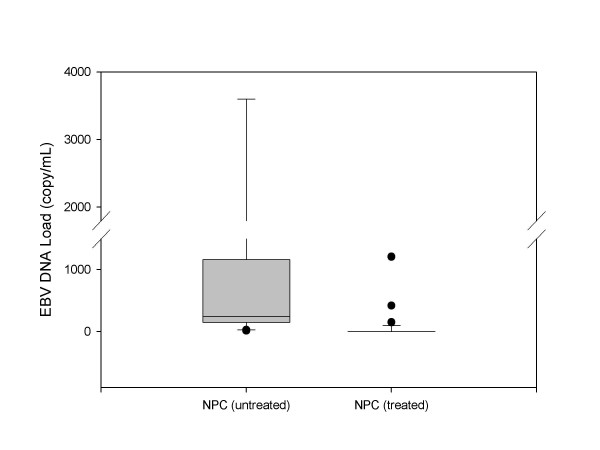
Box and whisker plot representing EBV DNA load in 34 NPC patients who experienced significant decrease in plasma viral DNA load following treatment (*t*(33) = 2.566, p < 0.05). Pre-treatment EBV DNA load is 1669 ± 637 copies/mL (median = 243 copies/mL) as compared to 57 ± 37 copies/mL post-treatment (median = 0 copies/mL).

A comparison was made between the plasma EBV DNA load in 51 NPC patients before and following treatment (Table 1). Of the 51 patients, 34 patients showed significant reductions in viral DNA load following treatments. A mean DNA load of 1669 ± 637 copies/mL was detected before treatment and was reduced to 57 ± 37 copies/mL after treatment [*t*(33) = 2.566, p < 0.05] (Figure 1). In eight of the 51 patients, increases in EBV DNA loads following treatments were noted. However, the difference in mean viral loads before (559 ± 436 copies/mL) and after (13600 ± 12773 copies/mL) treatment in these eight patients was statistically insignificant due to large standard variation. Interestingly, the remaining nine patients had undetectable plasma EBV loads both before and after treatment. All the 39 healthy controls had undetectable plasma EBV load.

Next, we assessed the relationship between the stages of tumour and plasma EBV load. EBV load was quantified in the plasma samples of 78 untreated NPC patients comprising of five stage I, 19 stage II, 15 stage III, and 39 stage IV. Regression analysis failed to show significant associations between these two variables indicating that the plasma EBV DNA load was not a marker of tumour stage [*r*^2 ^= 0.012; *F*(1,76) = 0.935, p = 0.337]. The 78 NPC samples were then grouped according to their IgA-VCA titres, and the plasma EBV load was determined for each titre group. There was also no association between plasma IgA-VCA titre and EBV load [*r*^2 ^= 0.004; *F*(1,71) = 0.281, p = 0.598].

### Plasma cytokines levels, tumour stages and IgA-VCA titres in untreated NPC patients and following treatments

**Figure 2:**
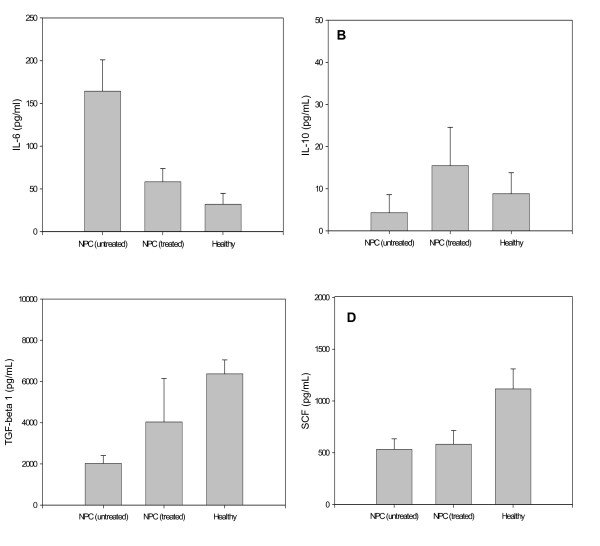
Plasma levels of (A) IL-6, (B) IL-10, (C) TGF-β1 and (D) SCF in 51 NPC patients, both before and after treatment, and 39 healthy controls. Quantification of cytokines and growth factors was performed using the BD OptEIA ELISA Set (BD Biosciences, USA). Bars indicate means and SE. Differences in means that are of statistical significance are as explained in the text.

The plasma levels of cytokines and growth factors – IL-6, IL-10, TGF-β1 and SCF in NPC patients, both before and after treatments, were determined using quantitative capture ELISA (Table 1). The cytokines, IL-6 and IL-10 have been widely investigated in solid tumours as being important prognostic factors [10]. Untreated NPC patients had a mean IL-6 concentration of 164 ± 37 pg/mL, which was significantly higher than the mean value following treatment (58 ± 16 g/mL; *F*(2,138) = 7.534, p < 0.01). The mean IL-6 concentration in NPC patients after treatment compared favourably with the level detected in healthy controls (mean = 32 ± 13 pg/mL; Figure 2A). There were no observable variations in the levels of plasma IL-10 between NPC patients, treated or otherwise and healthy controls (Figure 2B).

The level of TGF-β1 in untreated NPC patients (2020 ± 384 pg/mL) was found to be significantly lower than the healthy controls (6368 ± 682 pg/mL; p < 0.05). The same patients recorded an increase in mean TGF-β1 following treatment (4034 ± 2110 pg/mL; Figure 2C) although the difference does not differ significantly from the level before treatment (at α = 0.05).

The expression SCF or steel factor (*Sl*) receptor, c-kit, has been reported in NPC patients and is known to be confined only to the EBV-positive tumour cells [11]. SCF was found to be reduced in untreated NPC patients (532 ± 102 pg/mL) as compared to the level in healthy controls (1115 ± 193 pg/mL; *F*(2,138) = 4.823, p = 0.009). The level of SCF among the controls in this study compares favourably with the level of 1565 ± 352 pg/mL reported by Tao and co-workers [12] in their cohort of healthy individuals. There was no significant change in SCF level among the same NPC patients following treatment (581 ± 315 pg/mL; p = 0.963; Figure 2D).

When the level of each cytokine and growth factor was compared against the patients' IgA-VCA titres, no statistically significant association was found indicating that the plasma concentrations of IL-6, IL-10, TGF-β1 and SCF were independent of VCA antibody titres. Similarly, none of the cytokines tested showed any association with the stages of tumour.

### Plasma EBV load and cytokines levels

None of the cytokines tested had any significant correlations with plasma EBV DNA load in the 75 untreated NPC patients. Data from the other three patients were unavailable as their plasma samples were insufficient. However, among 51 of the 75 NPC patients who had been treated, significant positive correlations were found between EBV load and IL-10 [*r*(49) = 0.535, p < 0.01], IL-6 and IL-10 [*r*(49) = 0.474, p < 0.01], and between TGF-β1 and SCF [*r*(49) = 0.464, p < 0.01]. None of these correlations was observed either in the untreated NPC patients or in the healthy controls. The correlation between plasma IL-6 and IL-10 levels was also demonstrated in patients with non-Hodgkin's lymphoma (El-Far et al., 2004) and post-transplant lymphoproliferative disorder [13].

### IgA-VCA, EBV load and their clinical correlations

Five of the 78 NPC patients consented to have their blood samples taken during their initial presentations before receiving any form of treatments and at each subsequent visit when they returned to be treated. Treatments typically consisted of radiotherapy and/or chemotherapy (5-FU and cisplatin). Two of the five patients (NPC18 and NPC22) were presented with stage IIB tumours and had persistently high IgA-VCA titres and high plasma EBV load. A total of eight and six consecutive samples were collected from NPC18 and NPC22 respectively. Patient NPC18 had IgA-VCA titre of 80 before treatment and fluctuated between 40 and 80 during his subsequent visits. This patient also had detectable EBV load of 195 copies/mL during his initial presentation but escalated to a few thousand-fold peaking at 406,000 copies/mL in one of the subsequent visits. Patient NPC22 had similar IgA-VCA and plasma EBV load profiles as NPC18 but the EBV load peaked at 52,000 copies/mL in one subsequent visit. Both patients succumbed to their diseases and died due to disease progression.

In another two patients, NPC41 and NPC80, who were presented with tumour grades IVB and IIB respectively, persistently high IgA-VCA titres during their initial presentations and in the consecutive samples were detected. The IgA-VCA titres fluctuated between 40 and 80 in patient NPC41 but maintained at 80 throughout in patient NPC80. Interestingly, patient NPC41 recorded a very low plasma EBV DNA load during his first presentation (74 copies/mL) and had undetectable viral load during subsequent treatment work-ups. On the other hand, patient NPC80 had undetectable viral DNA load during his first presentation and in subsequent visits. In all, four consecutive samples each were collected from NPC41 and NPC80. Both patients survived and responded well to chemotherapy.

The remaining patient was presented with tumour stage III and showed neither elevated EBV antibody (IgA-VCA titre = 5) nor detectable plasma EBV load before and during his three subsequent visits. This patient also responded well to chemotherapy and survived. These findings confirm earlier reports that patients with persistently detectable plasma EBV DNA had worse survival than patient with low or undetectable viral DNA following therapy and that the cell-free EBV DNA was deemed a good prognosticator of treatment outcome [14,15].

## Discussion

To date, this is the first reported study that compares the plasma levels of EBV DNA, interleukins and growth factors in consecutive samples of NPC patients before and after receiving treatment. Untreated NPC patients typically had elevated IgA-VCA titres although the titres normally span a broad spectrum. Although the EBV-specific antibody is known to be an accurate indicator for the presence of disease, the current data failed to show any association of this parameter with the tumour stages. Over the past few years, cell-free EBV DNA has been increasingly recognized as a good prognostic marker especially for the monitoring for disease recurrence in NPC patients [6,14]. As shown in Figure 1, high EBV DNA load was frequently detected in plasma of untreated NPC patients and a majority of the patients (34/51) experienced a significant decline in viral DNA following therapy. Although, regression analysis did not show any association between plasma EBV DNA load and the stages of NPC at presentation, viral DNA load in addition to the IgA-VCA titre was found to be a good prognosticator for subsequent clinical event. This was demonstrated in the four of the five patients who consented to participate in our follow-up study. Two patients who were presented with stage IIB disease and had persistently high viral DNA load but wavering IgA-VCA titres during the course of treatments died of disease progression. On the other hand, two other patients presented with stages IVB and IIB respectively, who had persistently high IgA-VCA titres but low and detectable plasma viral DNA survived. Moreover, favourable treatment outcome was demonstrated in the remaining patient presented with stage III disease who had neither detectable plasma EBV DNA nor IgA-VCA titre. These clinical observations underscore and confirm the disease prognostic value of cell-free EBV DNA in agreement with the earlier reports by Lo and co-workers [6] and more recently by Lin and co-workers [15].

The significant reduction in plasma IL-6 level and EBV DNA load suggest a state of inflammation that was most probably triggered by EBV infection of tumour cells. This is in line with a recent study by Chow and co-workers [16] which shows a correlation between elevated IL-6 levels in serum as well as in tumour tissues of NPC patients. The reduction of post-treatment IL-6 level was correlated to a slight but statistically insignificant increase in IL-10 levels (Table 1). IL-10 is known for its role in countering and limiting the extent of inflammation following tissue injury or infections [17]. Although IL-10 was reported to be expressed in tissues of primary NPC [8,18], our data did not indicate any observable differences in the level of this interleukin in the plasma of NPC patients in comparison to controls. This suggests that the expression of IL-10 is confined to the tumour microenvironment and is not secreted. IL-10 expression thought to be a mechanism by which EBV-infected NPC cells counter the local immune defense by reducing the number of cytotoxic T-cells in the tumour microenvironment [18]. This hypothesis can be corroborated with our current data indicating a post-treatment positive correlation between IL-6 and IL-10 levels where increased level of IL-6 was countered by the proportionate increases in IL-10. IL-10 expression in NPC tissues [8] and high IL-10 level in serum of PTLD patients [10] have been shown to be associated with poor survival. These findings are supported by our current data that indicate a strong correlation between plasma IL-10 level and EBV DNA load observed in NPC patients following treatment. As has been discussed, high plasma EBV DNA, like IL-10, is associated with poor prognosis and survival in NPC.

Of particular interest was the observation that NPC patients, treated or otherwise, showed a lower level of plasma TGF-β1 levels as compared to the healthy controls, suggestive of a protective role for this growth factor in NPC. Although elevated serum levels of TGF-β1 in NPC patients has been reported in one study [7], the tumour-suppressive effects of TGF-β1 has to taken into consideration. Over the recent years, TGF-β has been increasingly recognized as a potent tumour suppressor in several epithelial and other cell types [19,20]. To date, TGF-β, is known to for its ability to act as both a tumour suppressor as well as a tumour promoter. The tumour suppressive effect is especially pronounced during the early benign stage but genetic and biochemical alterations during tumorigenesis may subvert the role of TGF-β to predominantly tumour promotion. Thus, our data which indicates a significant decrease in the levels of this growth factor in NPC patients and a slight recorded increase following treatment is in favour of the tumour suppressive role of TGF-β.

Recently, the expression of c-kit and its corresponding ligand, SCF, have been shown in NPC cells [11,21]. SCF has diverse roles in haematopoiesis [22], angiogenesis [23] and apotosis [24] and affects the growth and differentiation of a vast range of cells. While no significant changes in SCF levels were observed in the NPC patients before and after treatment, a strong correlation between the level of this growth factor and the level of TGF-β1 was noted in patients after treatment. This is of particular interest as TGF-β1 is known to counter the cellular proliferative effects of SCF [25]. A longitudinal study is being planned to relate plasma TGF-β1 and SCF levels to clinical outcomes in NPC patients. A corresponding recent study has indicated that mast cells infiltration into nasal polyp tissue is dependent on SCF secreted by the epithelial cells [26]. This warrants further investigations on the involvement SCF-dependent cells of the immune system for example, the mast cells, in the pathogenesis of NPC.

## Conclusion

To this end, we conclude from our present data that plasma EBV DNA load is an excellent indicator for the presence of NPC and a good prognosticator for disease progression and clinical outcome. Elevated EBV DNA in the plasma was shown to be correlated to IL-10 in NPC patients after treatment. The significant reduction in plasma EBV DNA load and IL-6 levels in NPC patients following treatment is suggestive of positive response to treatment. The reduction in plasma TGF-β1 and SCF levels observed in NPC patients in comparison to controls warrants further investigations into the roles of these growth factors in the pathogenesis of NPC.

## Competing interests

The author(s) declare that they have no competing interests.

## Authors' contributions

All authors read and approved the final manuscript.

TEL: Responsible for the design and conduct of experiments, data analysis and preparation of manuscript.

SG and KR: Responsible for patient recruitment, blood sample collection, analysing data and aided in the interpretation of results.

SCK: Ensured approval from the University's Medical Ethics Committee and reviewed final manuscript submission.

## Pre-publication history

The pre-publication history for this paper can be accessed here:



## Figures and Tables

**Table 1:** Comparison of mean plasma EBV DNA load and cytokine levels between NPC patients and controls.

**Plasma Factors**	**NPC Patients (mean ± SE)**		**Healthy Controls (mean ± SE; n = 39)**
		
	**Untreated (n = 51)**	**Treated (n = 51)**	
EBV Load (copy/ml)	1669 ± 637	57 ± 37	0 ± 0
IL-6 (pg/ml)	164 ± 37	58 ± 16	31 ± 13
IL-10 (pg/mL)	4 ± 4	15 ± 9	9 ± 5
TGF-β1 (pg/mL)	2020 ± 384	4034 ± 2110	6368 ± 683
SCF (pg/mL)	532 ± 102	581 ± 133	1115 ± 193

Plasma samples from NPC patients, both pre- and post-treatment, and healthy controls were analyzed for the EBV DNA load, IL-6, IL-10, TGF-β1 and SCF levels. Data represents mean ± SE. Differences in means that are of statistical significance are explained in the text.
